# Comparison of adaptations to four weeks of continuous hypoxic training between healthy men with lower and higher initial aerobic fitness

**DOI:** 10.3389/fphys.2026.1880404

**Published:** 2026-07-15

**Authors:** Limingfei Zhou, Haitao Zhu, Luyu Zhang, Fang Zhou

**Affiliations:** 1 School of Strength and Conditioning Training, Beijing Sport University, Beijing, China; 2 Key Laboratory for Performance Training and Recovery, General Administration of Sport of China, Beijing, China; 3 Division of Sports Science and Physical Education, Tsinghua University, Beijing, China

**Keywords:** aerobic fitness, muscle oxygenation, normobaric hypoxia, ventilatory threshold, VO2peak, Yo-Yo intermittent recovery test

## Abstract

**Background:**

Continuous hypoxic treadmill training increases the physiological load of aerobic exercise, but it remains unclear whether baseline aerobic fitness influences short-term adaptation. This study compared the responses of healthy men with lower and higher initial aerobic fitness to four weeks of continuous treadmill training performed in normobaric hypoxia equivalent to a simulated altitude of 2500 m.

**Methods:**

Twenty-two healthy adult men were classified into lower- group (LF) and higher-fitness groups (HF) according to baseline treadmill graded exercise test performance. All participants completed the same four-week hypoxic treadmill training program and the same pre- and post-intervention tests, including a graded treadmill test with cardiorespiratory and oxygenation monitoring and a Yo-Yo Intermittent Recovery Test Level 1 (Yo-Yo IR1).

**Results:**

Both groups improved after training, but the changes were consistently larger in the LF group. Compared with the HF group, the LF group showed larger percentage improvements in relative VO__2_peak_ (+8.5% vs. +0.7%), peak treadmill speed (+9.9% vs. +1.6%), time to exhaustion (+10.4% vs. +2.3%), VT2 speed (+14.0% vs. +2.0%), percentage of VO__2_peak_ at VT2 (+7.5% vs. +1.2%), and Yo-Yo IR1 distance (+23.7% vs. +4.2%). Selected oxygenation variables at VT2 and during early recovery changed in the same direction, whereas minimum SpO_2_ and minimum SmO_2_ changed little.

**Conclusions:**

Four weeks of continuous hypoxic treadmill training were associated greater improvements in aerobic and threshold-related outcomes in men with lower initial aerobic fitness. The oxygenation outcomes provide descriptive physiological information only and should not be interpreted as evidence of hematological adaptation.

## 1 Introduction

Hypoxic training is commonly used to increase physiological strain during aerobic exercise and to enhance training adaptation under reduced oxygen availability (Millet et al., 2010; Treff et al., 2022). Whether initial aerobic fitness shapes adaptation to the same short-term continuous hypoxic treadmill training stimulus remains an important practical and scientific question. Continuous training in normobaric hypoxia provides a controlled model for applying sustained endurance stress under reduced oxygen availability, because exercise intensity, session duration, and inspired oxygen fraction can be standardized with good precision (Dufour et al., 2006; Kelly et al., 2021). Hypoxic and altitude-training studies have repeatedly shown, however, that responses to a given hypoxic stimulus are not uniform, and average group effects may obscure substantial inter-individual variability in adaptation (Friedmann et al., 2005; Bonafiglia et al., 2021; Mujika et al., 2024).

This issue is especially relevant because responses to exercise training vary substantially between individuals. Interindividual differences in trainability have been reported across cardiorespiratory and performance outcomes, and baseline training status is one factor that may influence the magnitude of adaptation to a given exercise stimulus (Bonafiglia et al., 2021; Noone et al., 2024). More broadly, hypoxic exposure and hypoxic intervention studies have involved participants with different training backgrounds, suggesting that baseline training status may influence both the physiological disturbance induced by hypoxia and the magnitude of subsequent adaptation (Martin and O'Kroy, 1993; Chapman, 2013; Boulares et al., 2025). In addition, prior altitude and hypoxia research suggests that baseline characteristics may modify the response to a given stimulus, including pre-existing hematological or physiological status and the relative challenge imposed by the intervention (Friedmann et al., 2005; Meyler et al., 2021; Bonafiglia et al., 2022; Mujika et al., 2024).

Individuals with lower initial aerobic fitness may have greater room for improvement and may experience a fixed hypoxic training stimulus as a larger relative stress. By contrast, individuals with higher aerobic fitness may require a greater training load, a longer intervention period, or a more individualized program to achieve additional gains. This pattern may also reflect differences in adaptation reserve. Lower-fitness individuals may have greater entry-level adaptation potential, whereas higher-fitness individuals may be closer to a performance ceiling, making the same short-term stimulus less likely to produce large additional gains (Zhou et al., 2025a, 2025). However, direct evidence comparing lower- and higher-fitness individuals during the same short-term continuous normobaric hypoxic treadmill program remains limited. Adaptation to continuous hypoxic training should not be evaluated through maximal aerobic capacity alone. Aerobic performance also depends on threshold-related exercise tolerance, graded treadmill-performance expression, and applied running-performance capacity (Westmacott et al., 2022; Yu et al., 2023). Yet adaptation to endurance-oriented hypoxic training is unlikely to be captured fully by maximal aerobic capacity alone, because it is also reflected in threshold-related responses and in how physiological function is sustained during progressive exercise (Contreras-Briceño et al., 2024; Benítez-Muñoz et al., 2025a). Cardiorespiratory adaptation can be assessed using direct laboratory measures, indirect performance-based measures, and physiological monitoring variables (American Thoracic and American College of Chest, 2003; Aandstad, 2021; Perrey et al., 2024). Direct gas-exchange assessment during graded exercise testing provides a standard laboratory approach for evaluating peak aerobic capacity and ventilatory-threshold responses, whereas field-based tests provide complementary information on functional running performance (Jones et al., 2016; Mayorga-Vega et al., 2016; Castro-Piñero et al., 2021; Dünnwald et al., 2021). Invasive measures, such as blood lactate, hemoglobin concentration, or hematological markers, can provide additional mechanistic information but increase participant burden and were not included in the present study (Brocherie et al., 2015; Cerezuela-Espejo et al., 2018; Yang et al., 2024). Non-invasive variables such as SpO_2_ and NIRS-derived SmO_2_ can provide contextual information on systemic and local oxygenation responses, but their reliability and interpretation depend on standardized measurement procedures, probe placement, adipose tissue thickness, and motion-artifact control (Poole and Jones, 2017; Sanni and McCully, 2019). Therefore, in the present study, oxygenation variables were treated as descriptive physiological measures rather than direct mechanistic endpoints.

Therefore, the present study compared the responses of healthy men with lower and higher initial aerobic fitness to four weeks of continuous normobaric hypoxic treadmill training at a simulated altitude of 2500 m. We hypothesized that the lower-fitness group would show larger pre–post changes, particularly in maximal aerobic capacity and threshold-related exercise tolerance. Graded treadmill and field-based running-performance outcomes were treated as complementary performance indicators, whereas SpO_2_ and SmO_2_ were considered supportive oxygenation context only.

## 2 Materials and methods

### 2.1 Participants

An *a priori* sample size calculation was performed using G*Power software (version 3.1; Heinrich-Heine-Universität Düsseldorf, Düsseldorf, Germany) (Faul et al., 2009). For a repeated-measures within-between interaction design, a minimum total sample size of 20 participants was required assuming a large effect size (f = 0.40), an α error probability of 0.05, a statistical power of 0.80, two groups, and two measurements. A large target effect size (f = 0.40) was specified *a priori* to support feasibility under the applied constraints of the study design. Given the applied nature of the study and the limited availability of eligible participants, the sample size calculation was used primarily to support study feasibility. Twenty-two healthy adult men volunteered and were therefore considered sufficient for the planned analyses according to the *a priori* calculation.

Participants were recreationally active and free from known cardiovascular, pulmonary, metabolic, or musculoskeletal conditions that would contraindicate maximal treadmill exercise according to standard exercise preparticipation screening principles (Thompson et al., 2013). None had completed systematic hypoxic training or been exposed to high-altitude environments (≥1000 m) during the 6 months before enrollment (Smith et al., 2022). Cardiorespiratory fitness strata were interpreted with reference to published directly measured VO__2_peak_ standards for healthy adult men from the FRIEND registry (Kaminsky et al., 2015, 2022). The FRIEND registry was used only to contextualize the cardiorespiratory fitness level of the participants. Group classification was based solely on baseline relative VO_2peak_ obtained from the treadmill graded exercise test. Self-reported fitness level was not used for group allocation. After baseline testing, participants were ranked according to relative VO_2peak_ and divided into LF and HF groups to create two clearly contrasting aerobic-fitness strata within the available sample. After baseline GXT, participants were ranked by relative VO_2peak_ and divided into lower- and higher-fitness groups to create two clearly contrasting aerobic-fitness strata within the available sample. The observed VO_2peak_ ranges were 37.7–46.1 mL·kg^-^¹·min^-^¹ in the LF group and 56.6–64.2 mL·kg^-^¹·min^-^¹ in the HF group. No participant in the recruited sample had a baseline VO_2peak_ between 46.1 and 56.6 mL·kg^-^¹·min^-^¹; this gap reflected the observed distribution of the available participants and was not created by excluding intermediate participants. All participants provided written informed consent before enrollment. The study was conducted in accordance with the Declaration of Helsinki and was approved by the Research Ethics Committee of Beijing Sport University (Approval No. 2023112H). Participants were instructed to avoid alcohol and to refrain from acute caffeine intake for at least 24 h before testing to minimize the acute ergogenic influence of caffeine on aerobic performance. Habitual caffeine intake was not formally quantified, which was acknowledged as a limitation. Participants were instructed to avoid vigorous exercise, defined as structured endurance, resistance, or high-intensity interval exercise perceived as ≥7 on a 0–10 RPE scale, for at least 24 h before each testing session. Compliance with these instructions was confirmed verbally before each session.

### 2.2 Study design

This study used a four-week, two-group, pre–post design. Participants completed baseline testing, were classified into LF and HF groups according to baseline relative VO__2_peak_, completed the same continuous normobaric hypoxic treadmill training program, and then completed the same post-intervention tests. Standardized verbal instructions and encouragement were provided using the same wording and timing for all participants, regardless of group. No additional motivational cues were provided specifically to LF participants.

Before baseline testing, participants were familiarized with the training and testing procedures. All pre- and post-intervention assessments were conducted in the same facility, at the same time of day for each participant, using the same instruments, assessors, and testing order. Participants were instructed to refrain from strenuous physical activity for at least 24 h before each testing session and to avoid alcohol and caffeine within 24 h of testing.

#### 2.2.1 Training protocol

Participants were instructed to avoid vigorous exercise, defined as structured endurance, resistance, or high-intensity interval exercise perceived as ≥7 on a 0–10 RPE scale, for at least 24 h before each testing session (Zhou et al., 2025b). All participants completed a four-week continuous normobaric hypoxic treadmill training program. Training was performed three times per week on nonconsecutive days. The hypoxic system used nitrogen dilution to reduce the fraction of inspired oxygen (FiO_2_), and environmental conditions were maintained at 22–25 °C and 40%–60% relative humidity throughout the intervention (Yan et al., 2021). Both groups trained in a hypoxic environment simulating an altitude of 2500 m (FiO_2_ = 14.8%–15.3%). A simulated altitude of 2500 m was selected to provide a moderate normobaric hypoxic stimulus that increases physiological strain during submaximal endurance exercise while remaining tolerable for repeated supervised training sessions in healthy adults. This altitude should be interpreted as the specific hypoxic dose used in the present protocol rather than as a universal or standard altitude for hypoxic training (Zhou et al., 2025b). Testing was conducted under controlled laboratory conditions, with temperature maintained at 22–25 °C and relative humidity at 40%–60%. Participants wore their own running shoes, but they were instructed to use the same shoes and similar lightweight athletic clothing at pre- and post-testing. All participants completed one familiarization session 48–72 h before baseline testing and before the first training session. The familiarization session included introduction to treadmill running under the testing protocol, NIRS and SpO_2_ device placement, standardized warm-up procedures, and practice of the Yo-Yo IR1 running pattern. Participants were also familiarized with the hypoxic treadmill training environment and safety-monitoring procedures. The same familiarization procedures were applied to all participants. In this study, the gas-exchange threshold (GET) was used to prescribe training intensity because it provides an individualized metabolic reference point that can be applied across participants with different aerobic-fitness levels. The prescribed intensity therefore referred to the treadmill running speed corresponding to each participant’s pre-intervention GET. VT2 was retained as a separate threshold-related outcome and was not used interchangeably with GET or anaerobic threshold.

Each session included a 45-min treadmill endurance run (h/p/cosmos, Nussdorf-Traunstein, Germany). Training intensity was prescribed as the treadmill running speed corresponding to the pre-intervention individualized GET. VO_2_ was measured during the graded exercise test and used to determine the individualized threshold-derived treadmill speed; it was not continuously measured during every training session. During training, the prescribed treadmill speed was used to control external workload, whereas HR and SpO_2_ were monitored continuously to supervise exercise tolerance, identify abnormal desaturation or excessive cardiovascular strain, and ensure participant safety during hypoxic exercise (Rogers et al., 2021; Faricier et al., 2024). HR and SpO_2_ were not used for real-time adjustment of treadmill speed. Cardiac drift was not used to modify training speed during sessions; therefore, the intervention should be interpreted as GET-derived external workload training rather than continuous physiological clamping exactly at the threshold. VT2 was determined separately as a threshold-related outcome during the graded exercise test. VO_2_ at VT2 was interpreted as a submaximal threshold-related response and not as a substitute for VO__2_peak_. VO__2_peak_ was defined as the highest 30-s averaged oxygen uptake value achieved during the test. Maximal effort was supported by volitional exhaustion and at least two of the following criteria: RER ≥ 1.10, HR within 10 bpm of age-predicted HRmax calculated as 208 − 0.7 × age, inability to maintain the required treadmill workload despite verbal encouragement, or a VO_2_ plateau if observed. Running speed was standardized by treadmill speed, but running cadence was not externally controlled or recorded. Before each session, participants completed a standardized warm-up including low-intensity running, coordination exercises, dynamic stretching, movement-integration drills, and neural activation. Throughout each session, SpO_2_ and HR (Polar 3.0, Finland) were continuously monitored. Heart rate was monitored continuously using a Polar heart-rate monitor. The device was not validated against ECG in the present study; therefore, HR was used for exercise monitoring and descriptive interpretation rather than as a primary outcome. After the training intervention, participants completed a standardized cool-down consisting of 8–15 min of static stretching. All sessions were supervised by certified strength and conditioning coaches to ensure consistent training execution and workload control. The facility was spacious, well-ventilated, and equipped with appropriate lighting, ensuring optimal conditions for the experiment.

#### 2.2.2 Outcome measurements

All participants completed the same measurements before and after the intervention. Testing sessions were conducted in the same facility, using the same instruments, assessors, and testing order at both time points. The assessment protocol included a treadmill graded exercise test with cardiorespiratory and oxygenation monitoring and a Yo-Yo intermittent recovery test level 1 as a complementary field-based measure of intermittent aerobic performance. The measurements included aerobic capacity, threshold-related exercise responses, field-based running performance, and descriptive oxygenation variables.

##### 2.2.2.1 Treadmill graded exercise test

A treadmill modality was selected because the intervention consisted of treadmill running and because the Yo-Yo IR1 also reflects running-based performance. Using the same locomotor modality for training and laboratory testing reduced modality-related variability and improved ecological consistency with the intervention. We acknowledge that VO_2_ values may differ between treadmill and cycle-ergometer protocols; therefore, the findings should be interpreted in relation to running-based exercise and should not be generalized directly to cycling or non-running exercise modalities. We used a treadmill graded exercise test to assess maximal aerobic capacity, graded exercise tolerance, and threshold-related responses (Miller, 2012). All tests were completed in the same laboratory under identical conditions before and after the intervention. After entering the laboratory, participants sat quietly for 20 min and then performed a 3-min warm-up at 7 km·h^-^¹. The test began at 8 km·h^-^¹, and running speed increased by 1 km·h^-^¹ every minute. Participants who reached volitional exhaustion before 16 km·h^-^¹ terminated the test before the incline stage. For participants who reached 16 km·h^-^¹, treadmill speed was maintained at 16 km·h^-^¹ and incline was then increased by 1% per minute until volitional exhaustion. This approach was used to maintain a progressive increase in exercise demand while avoiding excessively high treadmill speeds in fitter participants.

Respiratory gas-exchange variables, ventilation, and heart rate were monitored continuously using a metabolic cart (Moxus Metabolic Cart, AEI Technologies, USA) calibrated before each assessment according to the manufacturer’s instructions (Kabbadj et al., 2024). VO__2_peak_ was defined as the highest 30-s averaged oxygen uptake value obtained during the test, together with volitional exhaustion and supporting maximal-effort criteria (RER ≥ 1.10, HR within 10 bpm of age-predicted maximum, volitional exhaustion, plateau in VO_2_ if observed), consistent with established recommendations for VO__2_peak_ determination (Howley et al., 1995; Midgley et al., 2007). Peak treadmill speed and time to exhaustion were also retained for analysis because they provide practical information on performance during progressive treadmill exercise.

##### 2.2.2.2 Ventilatory threshold determination

We determined the second ventilatory threshold (VT2) from non-invasive gas-exchange and ventilatory criteria (Keir et al., 2022). GET was used for training-intensity prescription, whereas VT2 was retained as a threshold-related outcome. VT2 was identified using non-invasive ventilatory criteria, including the behavior of VE/VO_2_ and VE/VCO_2_, together with visual inspection of ventilation, oxygen uptake, carbon dioxide output, and respiratory exchange responses. Because blood lactate was not measured, VT2 was interpreted as a gas-exchange-derived ventilatory estimate rather than a lactate-validated metabolic breakpoint. Candidate criteria included the behavior of VE/VO_2_ and VE/VCO_2_ across the graded exercise test, together with visual inspection of ventilation, oxygen uptake, carbon dioxide output, and respiratory exchange responses. Because blood lactate was not measured, VT2 was interpreted as a reproducible ventilatory threshold estimate rather than a lactate-validated metabolic breakpoint. Thresholds were identified independently by trained assessors who were blinded to group and time point, and disagreements were resolved by consensus. VT2-related outcomes included running speed at VT2, VO_2_ at VT2, heart rate at VT2, percentage of HRmax at VT2, and percentage of VO__2_peak_ at VT2.

##### 2.2.2.3 Oxygenation measures

Muscle oxygenation was measured using a near-infrared spectroscopy (NIRS) system (Artinis Medical Systems, The Netherlands) controlled by Oxysoft software (version 3.0.103). The system employed dual-wavelength light sources at 760 nm and 850 nm to monitor changes in oxygenated and deoxygenated hemoglobin. The optode pair (light source and detector) was positioned over the belly of the vastus lateralis muscle at the midpoint between the greater trochanter and the lateral epicondyle of the femur. The interoptode spacing was 35 mm, which was selected to improve sensitivity to muscle tissue. Data were sampled at 10 Hz. Motion artifacts were corrected using spline interpolation and wavelet filtering, and a low-pass filter with a cutoff frequency of 0.5 Hz was applied to reduce high-frequency noise. NIRS measurements were conducted continuously during the entire graded exercise test and training sessions. The term ‘NIRS’ is used consistently throughout the manuscript to accurately reflect the measurement modality. NIRS data were recorded continuously during the graded exercise test. Before SmO_2_ assessment, all participants underwent skinfold thickness measurement at the vastus lateralis using a skinfold caliper. Skinfold thickness was assessed before NIRS probe placement. Because adipose tissue thickness may influence NIRS-derived muscle oxygenation signals, SmO_2_ outcomes were interpreted as descriptive and exploratory physiological variables rather than definitive mechanistic indicators. The evaluator carefully shaved the hair at the vastus lateralis region (10–12 cm superior to the lateral patellar border at the muscle belly) using a razor blade to ensure smooth skin surface for device placement. With the participant in a supine position, the evaluator exposed the target area and grasped the skin and subcutaneous tissue over the lateral thigh using the thumb, index finger, and middle finger. Subsequently, the skinfold caliper was applied 1 cm distal to the grasped tissue fold. Triplicate measurements were performed following standardized protocols, with either the median value or two consecutive matching readings recorded as the final measurement in millimeters (mm). For muscle oxygen saturation monitoring, the NIRS probe was secured perpendicular to the skin surface over the right vastus lateralis musculature (10–12 cm superior to the midpoint of the patella) using a light-opaque medical elastic bandage in crosswise configuration. The longitudinal axis of the sensor probe was carefully aligned parallel to the femoral shaft. Continuous data acquisition was performed at 10 Hz sampling frequency throughout testing.

##### 2.2.2.4 Yo-Yo intermittent recovery test

We used the Yo-Yo Intermittent Recovery Test Level 1 (Yo-Yo IR1) as a complementary field-based indicator of intermittent exercise performance with a strong aerobic contribution (Krustrup et al., 2003; Bangsbo et al., 2008). Before baseline testing, participants completed a familiarization session to standardize test procedures and reduce potential learning effects. The test was performed before and after the intervention in the same outdoor testing area, at the same time of day, according to standardized procedures. Participants completed repeated 2 × 20-m shuttle runs at progressively increasing speeds dictated by the official Yo-Yo IR1 audio recording, interspersed with 10 s of active recovery performed as a standard 2 × 5-m jog/walk around a marker, consistent with the original protocol. The test was terminated when a participant twice failed to reach the finish line in time, and the total distance completed (m) was retained for analysis.

### 2.3 Statistical analysis

The experimental data were processed using JASP (version 0.18.3, JASP Team, The Netherlands). Data are presented as mean ± standard deviation (SD) unless otherwise indicated. Normality was assessed using the Shapiro–Wilk test and visual inspection of Q–Q plots. Baseline between-group comparisons were performed for participant characteristics and baseline outcome values.

Primary and secondary outcomes were analyzed using two-way repeated-measures ANOVA with group (LF vs. HF) and time (pre- vs. post-intervention) as factors. The group × time interaction was the main inferential term because it tested whether pre–post changes differed between groups. Body mass was also analyzed using a two-way repeated-measures ANOVA because body mass can influence relative VO_2_peak. Absolute VO_2_peak was retained as a secondary outcome to help determine whether the observed pattern was limited to body-mass-normalized values.

For each outcome, absolute change, percentage change, 95% confidence intervals, within-group p values, within-group Cohen’s dz, between-group differences in change, and between-group effect sizes were reported where appropriate. Between-group effect sizes for change were calculated as the difference between LF and HF change scores divided by the pooled standard deviation of individual change scores. Effect sizes were interpreted as trivial (d < 0.2), small (0.2 ≤ d < 0.6), moderate (0.6 ≤ d < 1.2), large (1.2 ≤ d < 2.0), or very large (d ≥ 2.0). Because this standardization can yield very large values when change-score variability is small, effect sizes were interpreted together with raw changes, percentage changes, 95% confidence intervals, and group × time interaction effects.

Assumption checks were performed using Shapiro–Wilk tests, visual inspection, and Levene’s tests. Because some change-score variance assumptions were not fully satisfied, Welch tests and Mann–Whitney U tests were conducted as sensitivity analyses for between-group change comparisons. These sensitivity analyses did not materially alter the main pattern of findings. Statistical significance was set at p < 0.05.

## 3 Results

**Figure 1:**
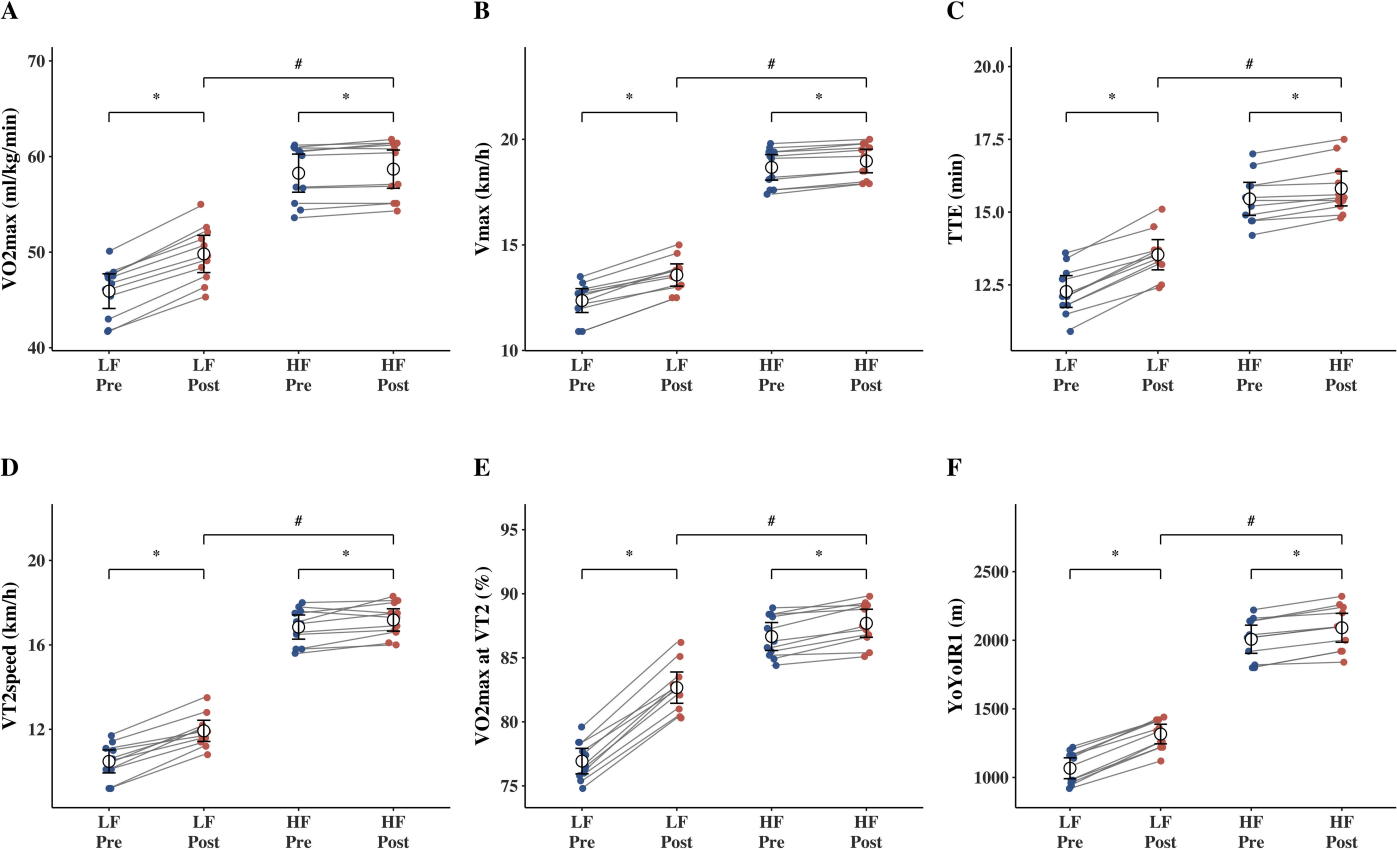
Primary outcomes and changes between pre- and post-intervention. **(A)** Relative VO₂peak; **(B)** peak treadmill speed; **(C)** time to exhaustion (TTE); **(D)** VT2 speed; **(E)** percentage of VO₂peak at VT2; **(F)** Yo-Yo Intermittent Recovery Test Level 1 (Yo-Yo IR1) distance. *p < 0.05, significant pre-to-post difference within the same group; #p < 0.05, significant between-group difference in change.

**Figure 2:**
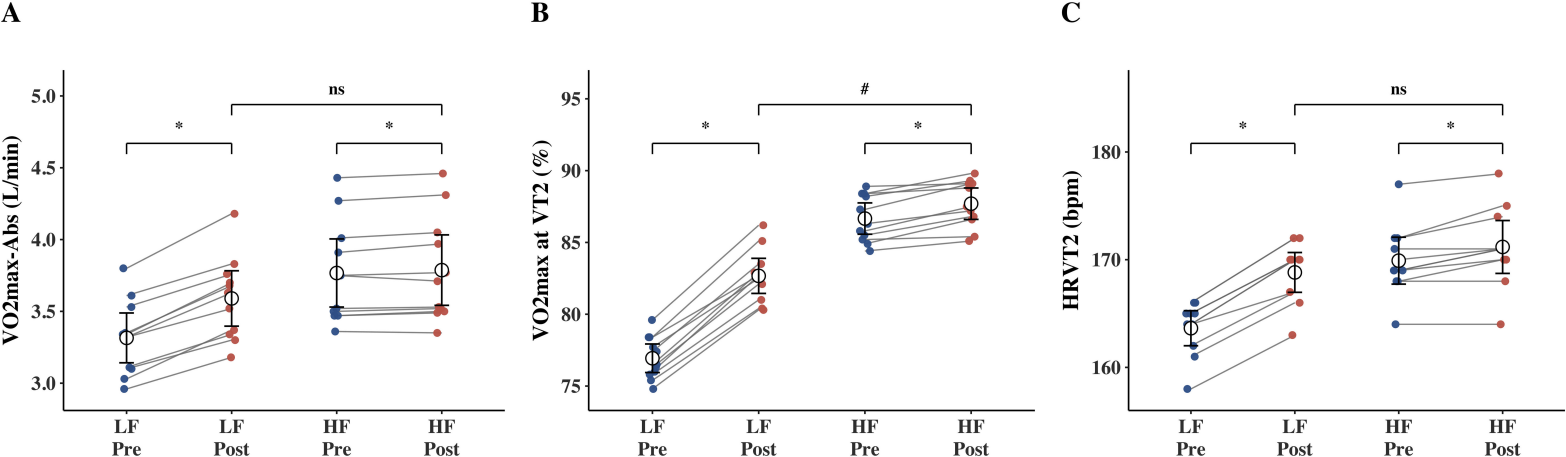
Secondary aerobic and cardiorespiratory outcomes. **(A)** Absolute VO₂peak; **(B)** VO₂ at VT2; **(C)** heart rate at VT2. *p < 0.05, significant pre-to-post difference within the same group; #p < 0.05, significant between-group difference in change; ns, non-significant difference.

**Figure 3:**
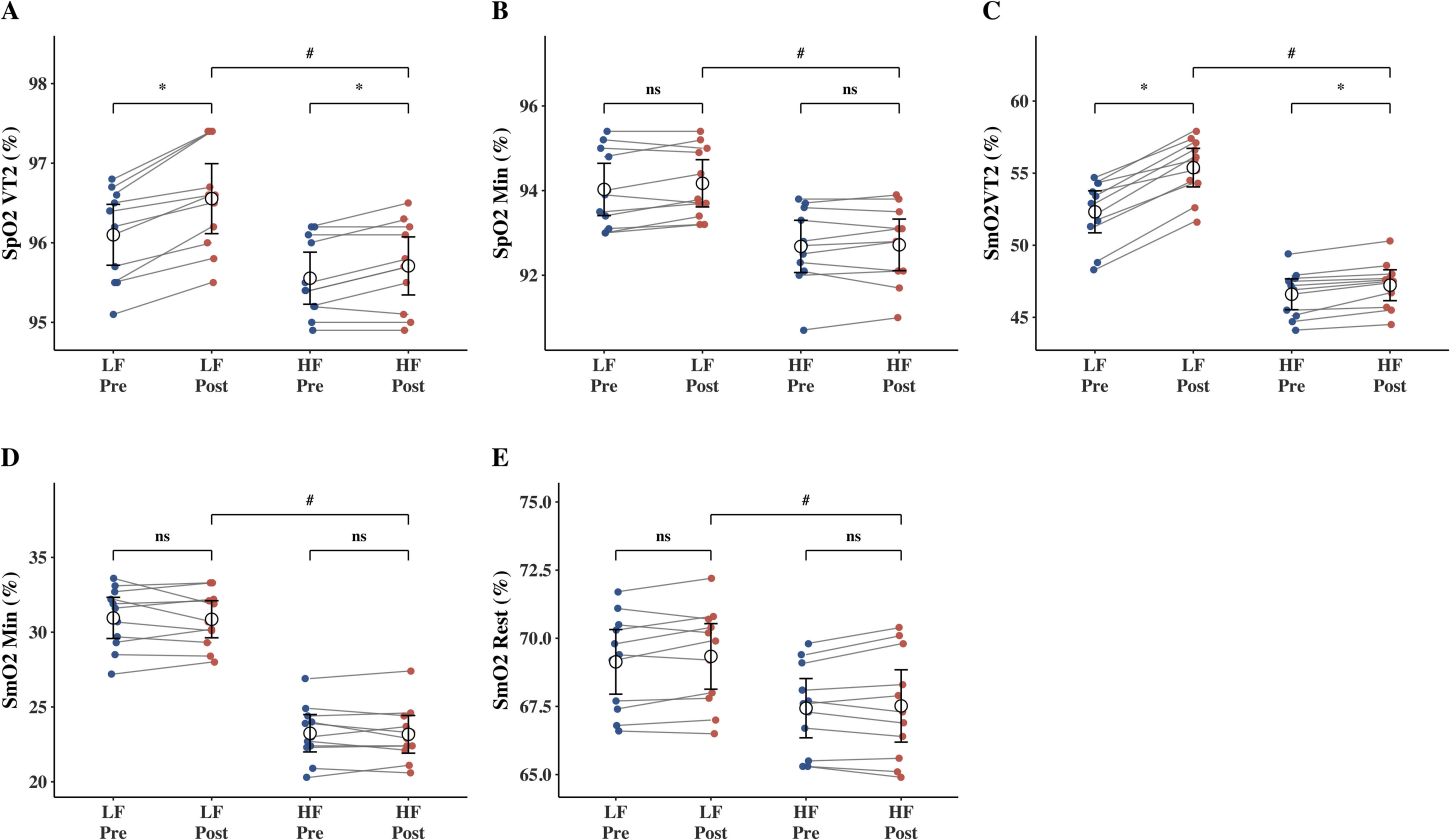
Oxygenation outcomes. **(A)** SpO₂ at VT2; **(B)** minimum SpO₂; **(C)** SmO₂ at VT2; **(D)** minimum SmO₂; **(E)** SmO₂ during the first minute of recovery. *p < 0.05, significant pre-to-post difference within the same group; #p < 0.05, significant between-group difference in change; ns, non-significant difference.

Descriptive statistics (mean ± SD) are presented in **Tables 1**–**4**. All 22 participants completed the study and were included in the final analysis. At baseline, one-way ANOVA showed no significant between-group differences in age, or height (*p* > 0.187). Individual responses and group-level changes for the primary outcomes, secondary aerobic and cardiorespiratory outcomes, and oxygenation outcomes are shown in **Figures 1**–**3**, respectively.

**Table 1:** Participant characteristics.

Variable	Lower aerobic fitness (n = 11)	Higher aerobic fitness (n = 11)
Age (y)	24.55 ± 3.30	24.82 ± 3.28
Height (cm)	174.37 ± 4.87	176.99 ± 4.09
Body mass pre (kg)	72.25 ± 4.56	64.58 ± 3.96
Body mass post (kg)	72.12 ± 4.65	64.47 ± 3.92
VO_2peak_ (mL·kg−1·min−1)	41.92 ± 2.70	61.26 ± 2.97
Absolute VO_2peak_ (L·min−1)	3.03 ± 0.25	3.96 ± 0.37

**Table 2:** Primary outcomes and change between pre- and post-intervention.

Outcome	LF pre	LF post	LF Δ [95% CI]	HF pre	HF post	HF Δ [95% CI]	Δ diff [95% CI]	p	d
VO_2peak_ (mL·kg−1·min−1)	41.92 ± 2.70	45.81 ± 2.90	3.89 [3.34 to 4.44]	61.26 ± 2.97	61.68 ± 2.98	0.42 [0.19 to 0.64]	3.47 [2.91 to 4.03]	<0.001	5.54
Peak speed (km·h−1)	12.36 ± 0.84	13.57 ± 0.78	1.21 [0.98 to 1.44]	18.67 ± 0.90	18.97 ± 0.83	0.30 [0.22 to 0.38]	0.91 [0.68 to 1.14]	<0.001	3.53
Time to exhaustion (min)	12.27 ± 0.81	13.54 ± 0.77	1.26 [1.03 to 1.50]	15.45 ± 0.85	15.81 ± 0.89	0.35 [0.21 to 0.50]	0.91 [0.65 to 1.17]	<0.001	3.09
VT2 speed (km·h−1)	10.48 ± 0.81	11.93 ± 0.75	1.45 [1.14 to 1.75]	16.85 ± 0.85	17.18 ± 0.79	0.34 [0.04 to 0.63]	1.11 [0.71 to 1.50]	<0.001	2.49
VO_2peak_ at VT2 (%)	76.95 ± 1.47	82.66 ± 1.79	5.71 [5.14 to 6.27]	86.67 ± 1.63	87.72 ± 1.60	1.05 [0.65 to 1.44]	4.66 [4.02 to 5.31]	<0.001	6.40
Yo-Yo IR1 (m)	1067.27 ± 113.23	1316.36 ± 106.52	249.09 [221.32 to 276.86]	2007.27 ± 152.91	2090.91 ± 156.55	83.64 [61.30 to 105.97]	165.45 [132.09 to 198.82]	<0.001	4.41

**Table 3:** Secondary aerobic and cardiorespiratory outcomes.

Outcome	LF pre	LF post	LF Δ [95% CI]	HF pre	HF post	HF Δ [95% CI]	Δ diff [95% CI]	t	p	d for between-group Δ
Absolute VO_2peak_ (L·min−1)	3.03 ± 0.25	3.30 ± 0.28	0.28 [0.23 to 0.32]	3.96 ± 0.37	3.98 ± 0.38	0.02 [0.00 to 0.04]	0.26 [0.21 to 0.30]	11.11	<0.001	4.74
VO_2_ at VT2 (mL·kg−1·min−1)	32.25 ± 2.25	37.88 ± 2.79	5.63 [5.01 to 6.25]	53.10 ± 2.83	54.11 ± 2.87	1.01 [0.68 to 1.34]	4.62 [3.96 to 5.27]	14.72	<0.001	6.28
HR_max_ at VT2 (%)	85.69 ± 1.47	88.46 ± 1.54	2.77 [2.45 to 3.09]	90.88 ± 1.10	91.48 ± 1.15	0.60 [0.38 to 0.82]	2.17 [1.81 to 2.54]	12.47	<0.001	5.32

**Table 4:** Oxygenation outcomes.

Outcome	LF pre	LF post	LF Δ [95% CI]	HF pre	HF post	HF Δ [95% CI]	Δ diff [95% CI]	t	p	d for between-group Δ
SpO_2_ at VT2 (%)	96.10 ± 0.57	96.55 ± 0.65	0.45 [0.31 to 0.60]	95.55 ± 0.49	95.71 ± 0.54	0.15 [0.04 to 0.27]	0.30 [0.13 to 0.47]	3.62	0.002	1.54
Minimum SpO_2_ (%)	94.03 ± 0.92	94.17 ± 0.83	0.15 [-0.02 to 0.31]	92.68 ± 0.92	92.72 ± 0.91	0.04 [-0.12 to 0.20]	0.11 [-0.10 to 0.32]	1.07	0.299	0.45
SmO_2_ at VT2 (%)	52.32 ± 2.17	55.38 ± 1.99	3.06 [2.48 to 3.65]	46.60 ± 1.59	47.23 ± 1.59	0.63 [0.35 to 0.90]	2.44 [1.83 to 3.04]	8.43	<0.001	3.59
Minimum SmO_2_ (%)	30.95 ± 2.05	30.86 ± 1.84	-0.09 [-0.69 to 0.50]	23.25 ± 1.86	23.17 ± 1.87	-0.07 [-0.48 to 0.33]	-0.02 [-0.69 to 0.66]	-0.06	0.956	-0.02
SmO_2_ 1-min recovery (%)	51.24 ± 2.59	54.46 ± 2.72	3.23 [2.52 to 3.93]	47.98 ± 2.38	48.80 ± 2.18	0.82 [0.42 to 1.21]	2.41 [1.65 to 3.17]	6.64	<0.001	2.83

### 3.1 Aerobic and exercise-performance

Two-way repeated-measures ANOVA revealed significant group × time interactions for all Aerobic and exercise-performance assessed by relative VO__2_peak_, peak treadmill speed, time to exhaustion, VT2 speed, percentage of VO__2_peak_ at VT2 and Yo-Yo IR1 distance between LF group and HF group. Bonferroni-adjusted *post hoc* analyses showed that relative VO__2_peak_ (95% CI [3.34, 4.44], adjusted *p* < 0.001, *d* = 4.74), peak treadmill speed (95% CI [0.98, 1.44], adjusted *p* < 0.001, *d* = 3.51), time to exhaustion (95% CI [1.03, 1.50], adjusted *p* < 0.001, *d* = 3.58), VT2 speed (95% CI [1.14, 1.75], adjusted *p* < 0.001, *d* = 3.23), percentage of VO__2_peak_ at VT2 (95% CI [5.17, 6.30], adjusted *p* < 0.001, *d* = 6.85), and Yo-Yo IR1 distance (95% CI [221.32, 276.86], adjusted *p* < 0.001, *d* = 6.03) were significantly greater after hypoxic training in LF group compared with HF group.

A significant improvements of relative VO__2_peak_ (Δ = 0.42 mL·kg^-^¹·min^-^¹, 95% CI [0.19, 0.64], *p* = 0.004, *d* = 1.24), peak treadmill speed (Δ = 0.30 km·h^-^¹, 95% CI [0.22, 0.38], *p* < 0.001, *d* = 2.54), time to exhaustion (Δ = 0.35 min, 95% CI [0.21, 0.50], *p* < 0.001, *d* = 1.61), percentage of VO__2_peak_ at VT2 (Δ = 1.04%, 95% CI [0.63, 1.44], *p* < 0.001, *d* = 1.73), and Yo-Yo IR1 distance (Δ = 83.64 m, 95% CI [61.30, 105.97], *p* < 0.001, *d* = 2.52) were observed in HF group after hypoxic training compared with pre intervention. In contrast, no significant increase was observed in VT2 speed in HF group after hypoxic training (Δ = 0.34 km·h^-^¹, 95% CI [0.04, 0.63], adjusted *p* = 0.060, *d* = 0.76).

### 3.2 Ventilatory-threshold-related outcomes

Two-way repeated-measures ANOVA revealed significant group × time interactions for all threshold-related outcomes assessed by absolute VO__2_peak_, VO_2_ at VT2, and percentage of HRmax at VT2 between LF group and HF group. Bonferroni-adjusted *post hoc* analyses showed that absolute VO__2_peak_ (95% CI [0.23, 0.32], adjusted p < 0.001, d = 3.96), VO_2_ at VT2 (95% CI [5.23, 6.48], adjusted p < 0.001, d = 6.30), and percentage of HRmax at VT2 (95% CI [2.48, 3.03], adjusted p < 0.001, d = 6.79) were significantly greater after hypoxic training in LF group compared with HF group.

Significant improvements of VO_2_ at VT2 (Δ = 0.96 mL·kg^-^¹·min^-^¹, 95% CI [0.65, 1.28], p < 0.001, d = 2.03) and percentage of HR_max_ at VT2 (Δ = 0.65%, 95% CI [0.35, 0.96], p = 0.002, d = 1.43) were observed in HF group after hypoxic training compared with pre intervention. In contrast, no significant increase was observed in absolute VO__2_peak_ in HF group after hypoxic training (Δ = 0.02 L·min^-^¹, 95% CI [0.00, 0.04], adjusted p = 0.066, d = 0.75).

### 3.3 Oxygenation outcomes

Two-way repeated-measures ANOVA revealed significant group × time interactions for oxygenation outcomes assessed by SpO_2_ at VT2, SmO_2_ at VT2, and SmO_2_ during the first minute of recovery between LF group and HF group. In contrast, no significant interaction effects were found for minimum SpO_2_ or minimum SmO_2_. Bonferroni-adjusted *post hoc* analyses showed that SpO_2_ at VT2 (95% CI [0.31, 0.60], adjusted p < 0.001, d = 2.10), SmO_2_ at VT2 (95% CI [2.48, 3.65], adjusted p < 0.001, d = 3.53), and SmO_2_ during the first minute of recovery (95% CI [2.52, 3.93], adjusted p < 0.001, d = 3.07) were significantly greater after hypoxic training in LF group compared with HF group.

A significant improvements of SpO_2_ at VT2 (Δ = 0.15%, 95% CI [0.04, 0.27], p = 0.026, d = 0.91), SmO_2_ at VT2 (Δ = 0.63%, 95% CI [0.35, 0.90], p < 0.001, d = 1.54), and SmO_2_ during the first minute of recovery (Δ = 0.82%, 95% CI [0.42, 1.21], p = 0.002, d = 1.39) were observed in HF group after hypoxic training compared with pre intervention. In contrast, no significant increase was observed in minimum SpO_2_ (Δ = 0.04%, 95% CI [-0.12, 0.20], adjusted p = 1.000, d = 0.16) or minimum SmO_2_ (Δ = -0.07%, 95% CI [-0.48, 0.33], adjusted p = 1.000, d = 0.12) in HF group after hypoxic training.

## 4 Discussion

The main finding of the present study was that four weeks of continuous normobaric hypoxic treadmill training at a simulated altitude of 2500 m elicited larger improvements in aerobic and threshold-related outcomes in healthy men with lower initial aerobic fitness than in those with higher initial aerobic fitness. This pattern was consistently observed across the principal exercise-performance variables, including relative VO_2peak_, peak treadmill speed, time to exhaustion, VT2 speed, percentage of VO_2peak_ at VT2, and Yo-Yo IR1 distance. Taken together, these findings indicate that baseline aerobic fitness meaningfully influenced the magnitude of adaptation to the present short-term hypoxic training stimulus. At the same time, this interpretation should remain cautious. As in other applied training studies, the present design does not allow a definitive mechanistic explanation, and the observed between-group differences should be understood as different response magnitudes under the same intervention rather than proof that hypoxic training is intrinsically effective only for lower-fitness individuals.

One possible reason for the larger improvement in the LF group is that the same hypoxic treadmill program represented a greater relative physiological challenge for participants with lower initial aerobic fitness (Takei et al., 2025). Individuals with lower baseline aerobic capacity may have had greater room for improvement and may therefore have responded more strongly to a fixed training dose performed under reduced oxygen availability. In contrast, the HF group may have required a greater overall training load, a longer intervention period, or a more individualized progression to induce comparable additional gains (Klügel et al., 2025). This interpretation is consistent with broader evidence showing that training responsiveness is influenced by baseline physiological status and that substantial inter-individual variability may remain even when participants receive the same exercise stimulus (Nolte et al., 2025). However, the present study also showed that the LF group was heavier than the HF group, and body composition was not assessed. Because the LF group also had higher body mass, the findings should not be interpreted as the isolated effect of aerobic fitness independent of body-size characteristics. Instead, the results should be understood as differences between the two observed fitness strata under the present study conditions. The larger adaptation in the LF group may reflect a combination of greater room for improvement, a larger relative physiological stress at the same absolute hypoxic exposure, and a lower ceiling for further adaptation compared with the HF group. For lower-fitness individuals, a fixed duration of treadmill running under hypoxia may impose a stronger cardiorespiratory and peripheral oxygen-utilization challenge relative to their initial capacity. In contrast, the HF group may have required a higher training volume, greater intensity progression, or longer exposure to induce comparable additional adaptations. These explanations remain speculative because direct measures of blood volume, hemoglobin mass, mitochondrial adaptation, or muscle oxidative enzyme activity were not obtained.

The aerobic and graded treadmill-performance outcomes provide the clearest support for this interpretation. Relative VO_2peak_, peak treadmill speed, and time to exhaustion all showed significant group × time interactions, and the magnitude of change was consistently greater in the LF group than in the HF group. This is important because it indicates that the training response was not limited to a single cardiorespiratory variable (Liu et al., 2025). Instead, the larger adaptation in the LF group was also expressed in how aerobic function translated into graded treadmill performance (Dufour et al., 2006; Deng et al., 2025). The increase in absolute VO_2peak_ in the LF group further strengthens this interpretation by showing that the pattern was not limited to body-mass-normalized outcomes. In contrast, although the HF group did exhibit small but significant increases in relative VO_2peak_, peak speed, and time to exhaustion, these changes were modest compared with those of the LF group. In practical terms, the same short-term continuous hypoxic program appeared sufficient to induce a robust aerobic stimulus in lower-fitness men, whereas it produced only limited additional adaptation in those who already had a high aerobic level (Takei et al., 2025).

The threshold-related outcomes provide an important submaximal perspective on the main finding. Compared with the HF group, the LF group showed larger increases in VT2 speed, VO2 at VT2, percentage of VO_2peak_ at VT2, and percentage of HR_max_ at VT2 (Keir et al., 2022; Parpa and Michaelides, 2025). These results suggest that, after training, the LF group was able to sustain a higher running speed and operate at a higher fraction of maximal aerobic capacity before reaching the second ventilatory threshold (Benítez-Muñoz et al., 2025a, 2025). This distinction is important because adaptation to endurance-oriented hypoxic training should not be interpreted through maximal aerobic capacity alone (Yu et al., 2023). In many applied settings, the ability to tolerate and express submaximal-to-high-intensity work is at least as relevant as a change in VO_2peak_ itself (Parpa and Michaelides, 2025). The present findings therefore suggest that the stronger response in the LF group was expressed not only at the maximal level, but also in improved threshold-related exercise tolerance (Benítez-Muñoz et al., 2025a). Nevertheless, these outcomes should still be interpreted within the limits of the current methodology (Keir et al., 2022). Because blood lactate was not measured, VT2 in the present study should be regarded as a reproducible gas-exchange-derived ventilatory estimate rather than a lactate-validated metabolic breakpoint (Contreras-Briceño et al., 2024).

The field-based running outcome showed the same directional pattern as the laboratory measures and therefore strengthens the applied relevance of the findings (Bucciarelli et al., 2026). Yo-Yo IR1 distance increased significantly in both groups, but the improvement was clearly larger in the LF group. This suggests that the larger laboratory-based adaptation in the LF group was accompanied by a meaningful enhancement in intermittent running performance outside the treadmill setting (Krustrup et al., 2003). Yo-Yo IR1 result should be interpreted as complementary rather than central evidence. The intervention consisted of continuous treadmill running rather than sport-specific intermittent conditioning, and the present study was not designed to determine whether hypoxic treadmill training is an optimal way to improve Yo-Yo performance per se (Faiss et al., 2013; Han and Liu, 2025). Rather, the Yo-Yo result is best viewed as additional support for the broader conclusion that the LF group derived greater functional benefit from the training program than the HF group (Xu et al., 2025).

The oxygenation findings should be interpreted more cautiously than the aerobic and performance results (Perrey et al., 2024; Sendra-Pérez et al., 2025). Significant improvements were observed for SpO_2_ at VT2, SmO2 at VT2, and SmO_2_ during the first minute of recovery, and these variables changed in the same general direction as the main performance outcomes, with larger responses in the LF group. This pattern suggests that the intervention altered oxygenation behavior at selected stages of graded exercise and early recovery (Kowalski et al., 2025). However, the oxygenation response was not uniform across all indices, as minimum SpO_2_ and minimum SmO_2_ showed no meaningful changes in either group. More importantly, these oxygenation variables should not be interpreted as mechanistic evidence explaining why the LF group improved more (Nolte et al., 2025). In the absence of blood-based measurements, they provide descriptive physiological context only and do not permit conclusions regarding erythropoiesis, hematological adaptation, blood-volume expansion, or lactate-related mechanisms. Thus, the oxygenation data are useful insofar as they show that selected exercise and recovery oxygenation responses shifted in parallel with the broader training response, but they should not be overextended beyond that descriptive role (Sendra-Pérez et al., 2025).

From a practical perspective, the present findings may be relevant to strength and conditioning coaches, sport scientists, and practitioners who use hypoxic environments for aerobic conditioning. The results suggest that healthy men with lower initial aerobic fitness may obtain larger short-term improvements from a fixed continuous hypoxic treadmill program than individuals who already have high aerobic fitness (Boulares et al., 2025; Takei et al., 2025). For fitter individuals, practitioners may need to consider a longer intervention, higher training volume, greater intensity progression, or more individualized hypoxic dose. These findings may also help researchers design future hypoxic-training studies by emphasizing baseline aerobic fitness as an important contextual factor. However, the present results should not be interpreted as a clinical prescription or as evidence of optimal hypoxic-training dosage (Dorelli et al., 2025).

Several considerations also temper the interpretation of the present discussion. First, the study used a two-group pre-post design without a non-training control group, so the results should be interpreted as differential response magnitudes rather than definitive evidence of intervention efficacy relative to no training. Second, the sample size was modest, which limits precision even though the effect pattern was consistent. Third, the LF group was heavier than the HF group, and body composition was not measured, which complicates interpretation of normalized physiological outcomes. Fourth, no blood-based markers were collected, limiting mechanistic inference. Because only healthy adult men were included, the findings cannot be generalized directly to women. Future studies should include female participants and consider menstrual-cycle phase, hormonal contraceptive use, and sex-specific responses to hypoxic exercise training. Blood-based measures such as hemoglobin concentration, hematocrit, blood volume, erythropoietin, ferritin, and blood lactate were not collected because the study was designed as a non-invasive applied training study with repeated exercise testing. Consequently, the present data cannot determine whether hematological adaptation, blood-volume expansion, erythropoiesis, or lactate-related mechanisms contributed to the observed changes. Even with these limitations, the present study provides coherent evidence that baseline aerobic fitness is an important contextual factor when interpreting short-term adaptations to continuous hypoxic treadmill training.

Overall, these findings indicate that baseline aerobic fitness influenced the magnitude of adaptation to this short-term continuous hypoxic training program. In this study, lower-fitness men exhibited consistently larger gains across the most relevant aerobic, threshold-related, and complementary performance outcomes, whereas oxygenation responses provided supportive but non-mechanistic physiological context. This pattern highlights the importance of considering initial fitness level when prescribing and interpreting hypoxic endurance-training interventions.

## 5 Conclusion

Four weeks of continuous hypoxic treadmill training at a simulated altitude of 2500 m were associated with larger improvements in aerobic and threshold-related outcomes in men with lower initial aerobic fitness than in men with higher initial aerobic fitness. The field-based running result supported the same pattern, whereas the oxygenation outcomes provided descriptive physiological context only. Overall, these findings indicate that baseline aerobic fitness influenced the magnitude of adaptation to the present short-term continuous hypoxic training program.

## Data Availability

The raw data supporting the conclusions of this article will be made available by the authors, without undue reservation.
